# HIV/AIDS activism and the Unified Health System in Belém, Brazil, 1990-2003

**DOI:** 10.1590/S0104-59702025000100022en

**Published:** 2025-05-19

**Authors:** Paulo Henrique Souza dos Santos, Carlos Henrique Assunção Paiva

**Affiliations:** i Doctoral candidate, Graduate Program in History of Science and Health/Casa de Oswaldo Cruz/Fiocruz. Rio de Janeiro – RJ – Brazil paulohen.rique@hotmail.com; ii Coordinator, Department of History of Science and Health Research/Casa de Oswaldo Cruz/Fiocruz. Rio de Janeiro – RJ – Brazil carlos.paiva@fiocruz.br

**Keywords:** Health history, Unified Health System (Sistema Único de Saúde – SUS, HIV/aids activism

## Abstract

The paper analyses, in historical perspective, the role of social organizations in combat against Aids as how it takes part in the process of institutional construction of the Sistema Único de Saúde (SUS) in the state of Pará, Brazil. Emphasizing the prominence of the group Paravidda, it discusses the demands for organization of hospital care from of SUS’s regulations in 1990 until 2003, when there was a restructuration of healthcare in region. It is concluded that Paravidda has become a prominent actor in regional implementation of SUS, meaning that organized civil society played a key role in the process of health reform and institutional construction of the SUS.

Laws passed in 1990, referred to as the Organic Health Laws (laws 8,080 and 8,142), provided the legal framework for the implementation of a new public health system, called the Unified Health System (Sistema Único de Saúde, SUS). This health system, which would assure all Brazilian citizens the right to health, providing comprehensive care in a decentralized manner, was to be developed with wide-ranging citizen engagement. These principles and guidelines were nothing new, insofar as such a system had been on the agenda of the discussions and struggles of different segments of society since the 1970s and 1980s, notably the public health movement, the people’s movement for health, and the feminist movement (Gerschman, 2004; Escorel, 2012).

The efforts to implement SUS in the 1990s, taking account of the interests of different institutions and social movements, nonetheless proved a challenging task (Faleiros et al., 2006; Gerschman, 2004; Paim, 2008). The prevailing neoliberal ideology, compounded by the programmatic agendas of subsequent neoliberal-oriented federal administrations, meant there was scant support for the social policies that had been envisaged. This political-ideological scenario constituted a major obstacle to the advancement of the health reform, resulting in undeniable impacts on the nation’s health (Paiva, Teixeira, 2014).

All of this clearly indicates that the institutionalization of SUS was in no way assured. Its regulation, in 1990, heralded a whole new cycle of social conflicts, which themselves reflected the political wrangles that had accompanied the drafting of the regulation (Faleiros et al., 2006; Gerschman, 2004; Paim, 2008). Indeed, the existence of well-coordinated, assertive health forums was fundamental in quashing President Fernando Collor de Mello’s veto of law 8,080/90, which established management bodies for societal participation, such as councils and conferences, at federal, state, and municipal level.

In the first decade after Brazil returned to democratic rule, as SUS was being formulated and implemented, one topic that fired heated discussions about sexuality and health was AIDS,^1^ especially in view of its impact on the homosexual community in Brazil (Aguiar Júnior, 2016; Daniel, Parker, 2018; Dias, 2012; Nascimento, 2005; Ramos, 2016; Santos, 2019; Vitiello, 2009). In the 1990s, homosexual men, lesbian women, female sex workers, bisexuals, transvestites, and health workers organized groups to form a consensus regarding the stigmatization of the disease and the lack of healthcare options for people living with HIV. These groups, based in different parts of the country, were instrumental in shaping debates and initiatives for the implementation of the new public health system.

In Belém, the state capital of Pará, AIDS also sparked public debate about stigmatization and access to healthcare (Santos, 2019). Two newspapers – *Diário do Pará* ^2^ and *O Liberal* ^3^ – became strategic vehicles for non-governmental organizations (NGOs) working with AIDS in Pará to publicize events and speak out about the health situation. Just as SUS was emerging, the Belém Homosexual Movement (Movimento Homossexual de Belém, MHB), the Pará Women Sex Workers Group (Grupo de Mulheres Prostitutas do Estado do Pará, Gempac), the AIDS Patient Support and Prevention Group (Grupo de Apoio e Prevenção do doente de Aids, Gapa-PA), and the Group for the Appreciation, Integrity, and Dignity of AIDS Patients (Grupo para a Valorização, Integração e Dignificação do Doente de Aids, Paravidda) joined forces in the fight for the constitutionally assured right to healthcare for people living with HIV.

In this context, the issues raised by members of Paravidda included the need for more hospital beds for the treatment of conditions associated with AIDS and the organization of the public healthcare network. These demands, made at the same time that SUS was being rolled out, reveal how this group engaged actively in the establishment of universal healthcare in Belém.

Despite the diversity of currents, focuses, and approaches in the historiography of AIDS in Brazil, it is striking how SUS is treated as almost incidental in these narratives (Aguiar Júnior, 2016; Barata, 2006; Barros, 2018; Galvão, 1997; Ramos, 2016; Santos, 2019; Souza, 2014; Teodorescu, Teixeira, 2015; Vianna, 2018). When it is mentioned, it is presented as a given, as established, existing in parallel to AIDS in the country. In this article, connections are drawn between the implementation of SUS and the different ways in which organized civil society, especially movements supporting people living with HIV, positioned itself in favor of the right to health.

Specifically, this article analyzes the demands for hospital care made by Paravidda in Belém from when SUS began to be rolled out until 2003, when the Ministry of Health published legislation (portaria 03/2003) making new hospital beds for AIDS patients available in Pará. Active from the 1980s onwards and formalized in 1992, the group was one of the pioneers in advocating for access to healthcare for people living with HIV in Belém.

One founding assumption of the analysis is that the sphere of public policymaking is not restricted to government actors, but is subject to interference from the broad spectrum of society. In this sense, civil society is considered a place where needs arise, which are sometimes aligned with organized groups devoted to communicating specific demands as part of a state that purports to be democratic (Bobbio, 2007).

Three sets of documents are analyzed in this study. The first set consists of the management reports of the Pará Department of Health.^4^ These reports set forth the main government initiatives related to the local health services, in line with the Basic Operating Standards (NOBs) for SUS, which set the criteria for decentralized, regionalized care.

The second group of documents consists of newspaper articles from *Diário do Pará* and *O Liberal*.^5^ These enabled the identification of different perspectives at play in the AIDS debate, while taking account of certain features of the publications, such as their particular interests and the political actors linked to them (Lucas, 2005). The news stories selected were the ones publishing complaints made by Paravidda to local health authorities and the ones reporting on local repercussions of the broader AIDS debate. Here, the press served as a forum through which Paravidda could direct its political demands to the health authorities.

The third set of documents consists of the testimony of key actors. These individuals’ statements reveal tensions and perspectives that are not apparent in the other documents, as they encompass multiple subjectivities and perspectives on the subjects of interest here (Voldman, 2006). Semi-structured interviews were held with these actors, most of whom were either HIV/AIDS activists linked to Paravidda at the time or people from Pará (state) or Belém (municipal) departments of health. The research was approved by the EPSJV/Fiocruz research ethics committee (approval 4,970,385).

The article is organized into three parts. The first discusses how local health authorities engaged in the development of the Unified Health System, SUS, in Pará state. The second focuses on the rise of HIV/AIDS activism in Brazil and Pará, as well as the political–institutional establishment of Paravidda. From this point on, the article explores the group’s demands for hospital care for people living with HIV as part of the development of a health system that was universal, decentralized, and participatory – the organizational and doctrinal pillars of SUS.

## Institutional developments: the construction of SUS in Belém, Pará state, in the 1990s

The challenges facing the implementation of SUS are expounded extensively in the Brazilian literature (Arretche, Marques, 2002; Cordeiro, 2004; Klein, 2011; Paim, 2008, 2009; Paiva, Fonseca, 2015; Silveira, Paim, Adrião, 2022). The first stage, which involved a range of issues, consisted of institutionalizing the operation of the health system to the municipal level (i.e., its decentralization) while also maintaining its unified nature, insofar as a single authority was established at each level of government (federal, state, and municipal). The unification of individual curative care, then the responsibility of the Ministry of Social Security, with prevention-oriented public health, then under the Ministry of Health, proved a major institutional challenge and one that was pivotal to the success of the health reform. From the 1990s onwards, under the Organic Health Laws, the challenge was how to roll out this unified system to the different states in the federation, including Pará, and thence to the municipalities, which, in the case of Pará, presented a whole host of specific challenges.

Pará is the second largest state in the North region of Brazil, covering 1,248,042km^2^, or 14.66% of the nation’s territory (Pará, 2023). It is part of the Amazon region, whose heterogeneous, multifaceted socioenvironmental characteristics range from densely populated conurbations to isolated towns, not to mention its great many different traditional populations and the large distances, with some municipalities situated more than 900km from their respective state capitals, making access to health services a challenge for much of the population (Garnelo, 2019; Kadri, Schweickardt, 2016; Oliveira, 2008; Nunes, 2018). Furthermore, the authorities often disregard regional hydrographic features, prioritizing land transport as the only way to cover great distances (Garnelo, 2019).

The demands for a healthcare network for AIDS in Pará were addressed amongst other issues related to the characteristics of the state and the dynamics of the administrative reorganization of the state and municipal departments of health (Pará, 1991a, 1991b, 1992, 1994, 1997, 1999, 2000). In the face of multiple conflicting political interests and social pressures, the restructuring of the local health institutions occurred in line with the NOB standards, published periodically by the Ministry of Health, which ultimately set the pace of the health reform (Arretche, 2005; Levcovitz, Lima, Machado, 2001; Scatena, Tanaka, 2001). It was in this context, in the second half of the 1990s, that the Healthcare Center for Acquired Infectious Diseases (Centro de Atenção à Saúde em Doenças Infecciosas Adquiridas, Casadia) was constructed in Belém.

In alignment with the government of President Fernando Collor de Mello, NOB-91 and NOB-92 were the first standards published with the aim of decentralizing the nascent SUS to municipal level – a political process supposedly in tune with the health reform. However, these standards established that the funds the municipalities would receive should be proportional to their pre-existing installed capacity and hospital and outpatient services, which meant municipalities with a better health infrastructure were disproportionately benefitted. Unsurprisingly, these rules came in for intense criticism from experts and social movements working in health, as not only did they exacerbate inequalities but the funds they allocated to state health departments to correct for such inequalities were inadequate (Costa, Silva, Ribeiro, 1999; Levcovitz, Lima, Machado, 2001; Scatena, Tanaka, 2001; Santos, Merhy, 2006).

Faced with these contradictions, the Pará Department of Health began efforts to coordinate the municipalities in the state for the decentralization of SUS. During the state administration of Jader Barbalho (1991-1994), the department also started to provide means for civil society participation and assistance for the municipal departments of health, establishing the State Municipalization Division, in March 1994, with a view to organizing the local management structures envisaged in NOB-93 (Pará, 1992, 1994).

NOB-93, published under President Itamar Franco, whose administration ran from 1992 to 1995, was designed to respond to demands for greater political-administrative autonomy for local SUS administrators.^6^ It established different classifications for the decentralized health administrations: incipient, partial, and semi-full for municipalities, and partial and semi-full for states. Each health administration was classified according to the level of complexity of the activities and functions the local managers could assume^7^ (Costa, Silva, Ribeiro, 1999; Levcovitz, Lima, Machado, 2001; Scatena, Tanaka, 2001).

These classifications required municipal and state departments of health to have compatible technical and administrative conditions. According to an assessment carried out when Almir Gabriel, from the Brazilian Social Democracy Party (Partido da Social-Democracia Brasileira, PSDB), was the governor of Pará, there were high rates of morbidity, a shortage of basic materials, devices, and medical and hospital equipment, buildings in a poor state of repair, and not enough professionals, especially in the interior of the state. In response, the state government called for greater coordination between state and municipal health institutions in order to ensure the local health system complied with the SUS guidelines (Pará, 1997).

As of the mid-1990s, as part of the reform agenda, the state took responsibility for specialized care and programs considered strategic. The Pará Department of Health’s regional centers were also earmarked for renovations and would take on responsibility for supporting the decentralization process in the municipalities under their jurisdiction. Furthermore, plans were made to transfer primary care services to the management of municipalities (Pará, 1997).

The Belém Department of Health followed these guidelines and its administration was visibly bolstered after NOB-93 and NOB-96 came into force. As a result, in 1996, it was classified as “semi-full” and, in 1998, it was promoted to “full” management of the entire municipal health system.^8^ From this point on, according to official documents, it had a fundamental role in the management and provision of services in the municipal and state health systems (Pará, 1997, 1998). It should be noted that NOB-96 only came into force in 1998, in the wake of debates and tensions under the Fernando Henrique Cardoso administration. This standard meant municipalities could be classified as having management of primary care only,^9^ or management of the entire municipal health system (Brazil, 5 nov. 1996).

If we observe the same institutional rollout of SUS in Belém and Pará through the lens of the local press, the story is less auspicious, with reports of numerous problems and conflicts. Particularly from 1997 onwards, during the administration of Mayor Edmilson Rodrigues, from the Workers’ Party (Partido dos Trabalhadores, PT), *Diário do Pará* published repeated complaints about the supposed malfunctioning of the city’s primary care facilities and the Municipal Accident and Emergency Hospital (Secretário..., 2 fev. 1997; Pronto-Socorro..., 4 mar. 1997; UMS..., 11 jan. 1997).

According to the local press, there were serious disputes between the municipal and state departments of health over the construction of a municipal hospital in the district of Guamá, which, according to the mayor, would relieve part of the demand for high-complexity care in the region (Secretário..., 21 out. 1998). As complaints mounted about the deteriorated state of the municipal hospitals and the care they provided, attention was drawn to the need for an effective pact that would enable the decentralization envisaged for SUS (Municipalização..., 5 ago. 1997; Secretary..., 1 jan. 1997; Sesma..., 7 jan. 1997).

By observing both sets of documents – the newspaper articles and the official documents – we can broadly identify two periods in the administrative reorganization of local institutions in Pará as part of the implementation of SUS. All data indicate that the Pará Department of Health made progress in putting the technical and administrative structure in place to support municipalities in the decentralization process between 1992 and 1996. From 1997 onwards, the Belém Department of Health began its reorganization to take over primary care facilities and municipal hospitals and to plan the expansion of health coverage in the city, including for AIDS.

Despite the wide range of financial and infrastructure needs and the countless wrangles and tensions spawned in the power-sharing process, especially when elected representatives and parties from different sides of the political spectrum were involved, it is fair to state that the region advanced in implementing the health system, largely due to the inductive and normative nature of the Ministry of Health and the administrative actions of the municipal and state departments of health. Nonetheless, as we will see below, this process also counted on organized civil society: its calls for health services and its political mobilization in a legal, political, and institutional context in which, for the first time, health was deemed a right of citizens in Brazil.

## HIV/AIDS activism: the creation of Paravidda in Belém in the 1990s

The AIDS epidemic posed challenges for federal, state, and municipal health administrators alike (Barros, 2018; Kadri, Schweickardt, 2016; Marques, 2002). Clinical knowledge about the disease was still incipient and the treatment options were largely ineffective. Furthermore, it was interpreted in some segments of society as being intrinsic to people deemed “promiscuous,” thus stigmatizing marginalized groups such as homosexuals, transvestites, bisexuals, and female sex workers (Dias, 2012; Gonçalves, 1989; Nascimento, 2005; Saldanha, 2023). This latter phenomenon hampered health initiatives for AIDS, since it was regarded in the public sphere as an individual problem rather than a public health issue, like any other condition.

In this context, several social organizations sprang up to call for healthcare for people living with HIV and challenge the associated stigmas. These organizations, referred to as AIDS NGOs, had multiple objectives: “Political activism, which called for the rights of patients, and solidarity, which sought to redefine the principles by which society addressed people infected with the virus” (Grangeiro, Laurindo da Silva, Teixeira, 2009, p.90).

The first home providing support for people with AIDS was founded in São Paulo in the 1980s by a transvestite activist, Brenda Lee. Its target public was transvestites and other people who were socially vulnerable because of discrimination related to AIDS, their sexual orientation, or their gender (Teodorescu, Teixeira, 2015). There were several other groups who also worked to defend the rights of people living with HIV in the 1980s and 1990s, such as the AIDS Prevention Support Groups (Grupos de Apoio de Prevenção à Aids – GAPA), the Brazilian Interdisciplinary AIDS Association (Associação Brasileira Interdisciplinar de Aids), featuring Herbert de Souza, and Pela Vidda, created by Herbert Daniel in 1989 (Barros, 2018; Contrera, 2000; Dias, 2012; Green, 2018; Vitiello, 2009; Zaquieu, 2006).

These and other entities began to organize meetings to discuss and align their positions on healthcare, such as the national meetings of AIDS NGOs (Galvão, 1997; Ongs/aids, 1989, 1990). With broad agendas, these meetings also revealed divergent opinions, especially on how people living with HIV were represented by others. This highly complex issue ultimately led to the formation of the National Network of HIV-positive People (Rede Nacional de Pessoas Soropositivas, RNP+), in 1995, which initially challenged the work and legitimacy of the AIDS NGOs for being overly bureaucratic and lacking in other ways (Câmara, Lima, 2000).

Even in the midst of so many tensions, these articulations, in dialogue with doctors, scientists, and individuals from the state apparatus, were important for the development of the National AIDS Program. Of equal important to this policymaking initiative were the AIDS NGOs’ actions and articulations in the development of programmatic agendas, the effective execution of actions and policies, and the production of concrete responses to the problems experienced by people living with HIV. Of particular note was the achievement of the universal distribution of medicines through SUS, starting in 1996 (Barros, 2018; Teodorescu, Teixeira, 2015).

As the national policies were being rolled out and more activist groups were formed around Brazil, the STD/AIDS Division of Pará was also created, with the participation of organized civil society, primarily Paravidda, which engaged in a range of discussion forums to demand universal hospital care in Belém – a fundamental principle of the nascent national health system.

Besides Paravidda, other groups also participated in discussions of the issues raised by AIDS in Belém, such as Gempac and Gapa-PA. The former was founded in the 1990s under the leadership of Lourdes Barreto, who had been instrumental in the creation of the Brazilian network of sex workers, Rede Brasileira de Prostitutas, in the 1980s, and in the fight for women’s rights, especially those of women sex workers in Pará and Brazil, since the 1990s. Gempac is a collective space that forged a consensus on the needs of female sex workers in Pará and also on sex education and disease prevention in policymaking forums for HIV/AIDS in the state, such as the state and municipal health council (Barreto, 2023; Saraiva, 2009; Teodorescu; Texeira, 2015). As for Gapa-PA, the group was idealized in the 1980s at the Federal University of Pará and was composed of homosexuals, students, and professionals from different areas with the aim of raising awareness about HIV/AIDS among the Pará population, reducing stigma, and fighting for the assurance of health services in the city (Martins, dez. 1990).

Paravidda was formed in the meetings at the AIDS Reference Unit to share information about HIV and support patients in Belém in the 1980s. It was only in the 1990s that its members gave it a name, with Augusto Marques, Luiz Antônio da Silva, and Maria Laurinda Silva Correia taking pivotal roles in its formation and continued existence (Correia, 16 set. 2021; Silva, 22 set. 2021; Paravidda, 1992a, 1992b).

Loss, suffering, and hope were what motivated these people to come together around the cause. The death of a son due to AIDS, for example, was a driving force behind Maria Laurinda’s trajectory. In her words, “I joined Paravidda because I had a foster child and he went away for a while and ... he came home contaminated ... So I started to take part, to study a lot, to find out what it was” (Correia, 16 set. 2021, p.1).

In the case of Luiz Antônio, a homosexual, it was testing positive for HIV that led him to a small support meeting at the AIDS Reference Unit, which he considered the kernel of the group. Every fortnight, on the premises of João de Barros Barreto University Hospital (henceforth Barros Barreto Hospital), he would talk about his experiences of living with the virus with a group of people (Silva, 22 set. 2021).

It is therefore necessary to recognize that the members of Paravidda went through different experiences with HIV before the group’s formalization. Maria Laurinda was sensitized by her son’s painful experience, while Luiz Antônio carried the virus and the social stigmas attached to it. According to its founders, the multiplicity of these experiences caused some friction within the group (Correia, 16 set. 2021; Silva, 22 set. 2021).

According to the minutes of the first official meeting, held on January 27, 1992, it was attended by ten people: Luiz Antônio da Silva, Maria Laurinda Silva Correia, Célia Maria Mattos da Cunha, Valdeliz Matos Teófilo, Ângela Maria Torres da Costa, Jorge Evandro de Amorim Matos, Silvia Helena Silva do Nascimento, Oswaldo Mattos, Jocélio Jorge, and José Carlos Nascimento (Paravidda, 1992b).

In 1994, Paravidda secured a building owned by the state government in Jurunas, a peripheral district of Belém, to house abandoned children living with HIV (Creche…, 15 nov. 1994). In addition, it also began to develop care initiatives for a wider public and provide temporary shelter for people from the interior of the state in clinical recovery or doing medical examinations (Paravidda, 1992c; Teodorescu, Teixeira, 2015). By the late 1990s, the group was appearing increasingly in discussions and demands for hospital care and in the construction of a state program for AIDS.

## Paravidda and hospital care: the fight for hospital beds in Belém and the institutional development of SUS, 1992-2003

As we have seen, the sheer size of Pará and its specific demographic and geographic characteristics make implementing public policies in the state a particularly challenging task. In the case of AIDS, these conditions imposed difficulties in access to hospital care, concentrated mostly at Barros Barreto Hospital, in Belém. This made developing policies for hospital care for people with the disease especially difficult.

Forums such as the municipal AIDS seminars, from 1992 onwards, were crucial in this process. Bringing together representatives from the Belém and Pará departments of health, the State Aids Division, the Municipal AIDS Commission, hospital directors, and representatives of NGOs in discussions about the disease, the seminars were where technical and political common ground was reached and, above all, where the interests and demands of organized civil society could be voiced. Paravidda took advantage of these seminars to discuss the health needs of people living with HIV from the perspective of the right to health and call for changes in two spheres: more health services and more disease prevention measures.

In the first two seminars, held in 1992 and 1993, Paravidda focused on the issue of healthcare, which it considered very restricted. Working with other actors, it was especially committed to getting a day hospital built, more beds in other SUS hospitals, and effective outpatient care, which would require the acquisition of medicines in sufficient quantity (Commission…, 5 dez. 1992).

Despite press reports of progress in AIDS care in Belém in the second half of the 1990s, there were still high levels of dissatisfaction among managers and activists about the state of affairs in the city. In 1997, for example, after Edmilson Rodrigues, from PT, took over as the mayor, Helena Brígido was appointed to the position of AIDS Coordinator. According to her, the Specialized Care Service was not running as it should: “In the year I joined the SAE [Specialized Care Service] ..., it was tiny compared to the AIDS Reference Unit. And the AIDS clinic was run there. ... We didn’t have enough room and it was harder to get patients into hospitals” (Brítido, 3 out. 2021, p.3). Another person who expressed reservations about the running of this service was Luiz Antônio do Paravidda: “Downstairs was a primary care unit and upstairs was for AIDS, but there was only one reception ... we had to discuss that. We needed a better space” (Silva, 22 set. 2021, p.8).

In view of these challenges, as of the mid-1990s Paravidda shifted its strategy of instigating public debate on the expansion of hospital care to a different arena. The first RNP+ meeting for the North of Brazil^10^ was organized by a committee composed of Antenor Chaves, Jaime Siqueira, Joaquim Barbosa, and Luiz Antônio da Silva with the aim of promoting the exchange of information among people living with HIV in the region (Portadores..., 4 mar. 1998; Portadores..., 20 mar. 1998). The event was attended by representatives from the states of Amazonas, Amapá, Acre, Rondônia, Roraima, and Pará, as well as Helena Brígido, in her role as municipal AIDS coordinator, health workers, and representatives from Paravidda, Gempac, Gapa-PA, and MHB. *O Liberal* wrote up the event, including issues raised by Luiz Antônio about the expansion of healthcare and the difficulties considered specific to the Amazon region. These are highlighted in the following passage:

Luiz Antônio da Silva says that carriers of the HIV virus who live in the Amazon do not have the same privileges as carriers living in the South and Southeast regions. Starting with the treatment provided by Sespa [Pará Department of Health] ... As in the last two years there has been an increase ... in the number of cases ... another reference unit specialized in AIDS … is needed (Encontro…, 16 mar. 1998, p.3).

It was in this context, under the leadership of Helena Brígido, that Casadia was created (completed in 1999), as an initiative of the municipal coordination team liaising with NGOs and with the support of the National AIDS Program. In the words of Brígido (3 out. 2021, p.3), “I drafted the Casadia project ... I went to Brasília ... for several meetings. ... We had frequent meetings with non-governmental organizations. ... Gapa, Paravidda, MHB. ... Paravidda was Dona Laura [Maria Laurinda]. We would get together and ask to create a document to forward to the [health] secretary.”

The reformulation of the Specialized Care Service and its expansion to incorporate the municipal outpatient clinic Casadia, in 1999, was also favored by the receipt of World Bank funds,^11^ through AIDS II,^12^ via the National AIDS Program, and by the strengthening of local health bodies. As mentioned, Belém had already been put in charge of primary care in the municipality in 1996 and had been promoted to management of the whole municipal health system in 1998, all of which indicates that the Municipal Department of Health had amassed sufficient institutional capacity to expand its services. In its administration of primary care, Belém had acquired a range of competences, including “the power to authorize the accreditation, de-accreditation, control, and evaluation of private and philanthropic hospital and outpatient services; [and] management of the outpatient network” (Costa, Silva, Ribeiro, 1999, p.39). Its status as the chief coordinator of the municipal system services was then reinforced in 1998, when it gained full management status.

We can see, therefore, that the opening of Casadia at the end of the decade was part of a broader process that involved close coordination among administrators and social movements, representing an undeniable institutional strengthening of the health authorities and service providers in the region. At the same time as this institutional progress was achieved, however, the local press reported that Barros Barreto Hospital was still the only healthcare facility where people living with HIV could receive care overnight and on weekends (Vem…, 22 set. 1999; Casa-dia…, 27 out. 1999).

Situations like this demonstrate that the response to the needs of people living with HIV depended crucially on the support offered by NGOs, as highlighted by Maria Laurinda Correia (16 set. 2021, p.11), from Paravidda: “If a patient was bad, I would send them to Casadia and they would spend the whole day there. The ambulance would come to pick them up. They would spend the whole day there on a drip including medication. At night Casadia would send them back to Paravidda and early in the morning they would go back.”

Such situations also demonstrate that implementing a single health system was neither simple, inevitable, nor free of contradictions. The question marks hanging over the process wherever there were liberal administrations and doctrines at play, not to mention the political and institutional hurdles, were counterbalanced, at least when it came to AIDS, by the conjunction of a few favorable circumstances: the national coordination of AIDS NGOs; the strengthening of the National AIDS Program; the institutional decentralization of SUS; and social participation in SUS, which enabled manifold opportunities for joined-up action in support of the health system.

As reported in *O Liberal* in March 2002, complaints about healthcare and pressure for its improvement, including legal action, were substantiated by the provisions of the 1988 Constitution, “among them, the requirement that establishments provide a certain number of beds to serve this public ... since article n.196 of the Federal Constitution establishes the assurance of equal and universal access to health actions and services as the duty of the state and the right of every citizen” (Falta…, 5 mar. 2002, p.7).

One problem that recurred in many other situations and in newspaper articles, and one that, over time, forged stronger ties between Paravidda, the Public Prosecution Service (Minisério Público), the Bar Association, and Gapa-PA, concerns the fight for better hospital care for people with HIV/AIDS-related medical needs, the success of which was in a constant state of flux. In November 2002, for example, *O Liberal* published a report about new hospital beds in Belém as of 2003: “Patients with AIDS will be able to receive treatment in two more hospitals in Belém, Santa Casa and Hospital das Clínicas, which will be making beds available from March 2003” (Santa…, 8 nov. 2002, p.5). Without doubt, this was a breakthrough

after a long, three-year struggle by the NGO Paravidda with the Public Prosecution Service and the Brazilian Bar Association. The director of Paravidda, Maria Laurinda Corrêa, said that three years ago she went to the Public Prosecution Service to report that hospitals were not providing care. ... ‘We also asked them to decentralize care from Barros Barreto [Hospital], because this was something the patients needed’ (Santa…, 8 nov. 2002, p.5).

In the early 2000s, the demands of social movements, as portrayed in newspaper articles, began to be included in discussions about the regionalization of health services and the organization of the healthcare network, as set forth in the new Healthcare Standards in 2001 and 2002. These standards led to efforts to promote greater region-level integration for health in the states, taking account of inequalities in access to care (Trevisan, Junqueira, 2007; Pereira, Quito, 2004).

The new Healthcare Standards put state executives in charge of developing regional planning actions for the network with the dual aims of making more cost-effective use of existing resources and ensuring better access to services by the population. To this end, the Regionalization Master Plan was drafted in a bid to organize the Brazilian states into healthcare districts and define care hubs and reference facilities. Ultimately, municipalities should work in a joined-up manner in the organization of healthcare, and each hub of a group of municipalities (healthcare district) should become a reference for a minimum set of healthcare services (Trevisan, Junqueira, 2007; Pereira, Quito, 2004).

In Pará, in order to take account of its great many mesoregions, internal inequalities, and the long distances between towns in the interior and the state capital – a reality encountered in other states – the plan was designed to reduce the dependence of smaller municipalities on Belém (Brasil, 1991; Pará, 2003a). In 2003, the year in which the Regionalization Master Plan for Pará was drawn up, there were, not surprisingly, high hopes that this would bring about an increase in the number of hospital beds for patients with HIV-related healthcare needs in the state (Saúde..., 3 jul. 2003).

Notwithstanding this institutional progress, the local press continued to fuel the debate and publish demands for improved access to hospital care (Treatamento…, 14 fev. 2003). Alongside other social movements, NGOs took the lead, spearheaded by Paravidda, which was frequently associated with the actions of the Public Prosecution Service to get new hospital beds made available in Belém and elsewhere in Pará. Under the new institutional framework, the provision of these beds for people with AIDS had to be organized in each of the nine health districts in the Regionalization Master Plan (Pará, 2003b).

Hospital beds would be set aside exclusively for AIDS-related complications if the municipal departments of health applied for them by the end of March 2003. The municipalities in charge of the whole municipal health system, including Belém, Ananindeua, and Castanhal, had to identify the hospitals, whereas municipalities in charge only of primary care relied on the state to make hospital beds available in state-managed hospitals (Pará, 2003b).

Against the odds, the SUS network of hospital care for patients living with HIV was gradually set up in the nine districts established in the Regionalization Master Plan as follows: In the metropolitan district, there were six beds in Ananindeua and forty-one in Belém, four of which were at Barros Barreto Hospital. In addition, there were two beds in Marituba and one each in Castanhal and Barcarena; in the Atlantic district, there was one bed at the Salinópolis Regional Hospital; in the Guamá district, there was one bed in Paragominas; in the Tocantins district, there was one bed in Tucuruí; in the Carajás district, there were two beds in Marabá; in the Xingu district, there was one bed at the Altamira Regional Hospital; in the Araguaia district, there was one bed in Redenção; in the Tapajós district, in view of the characteristics of the region and the access difficulties, two subdistricts were created, with two beds in Santarém and Itaituba; and in the Marajó district, considering the ease of access to Belém, hospitalizations and one dedicated bed should be provided by Barros Barreto Hospital (Pará, 2003b).

The same legislation that created these dedicated hospital beds indicated the need to plan for sixty-two new beds, which, according to the health secretary, Fernando Dourado, would represent not only an increase in the number of hospital beds in Belém, but also the “long-awaited decentralization of the care of these patients” (Tratamento…, 14 fev. 2003, p.8); in other words, the much-needed construction of a SUS care network in the region.

However, the volume of clinical emergencies was such that the institutional progress still fell short of meeting the needs of the state’s SUS users. For example, Paravidda made frequent complaints to the Municipal Department of Health about the chronic shortage of beds. Maria Laurinda Correia went so far as to say that the group received a constant flow of people from the interior of the state and also from the state capital in need of shelter and support while awaiting treatment. As she put it, “there are people who came here with a chance of recovery, but who took so long to get admitted that they ended up dying” (Leito…, 29 mar. 2003, p.7). In response, the Belém Department of Health reported that it had contacted the Pará Department of Health with requests for new hospital beds. The state authority would then have to forward these requests to the Ministry of Health so it could rubber stamp them through specific legislation (Leito…, 29 mar. 2003).

In view of this, the Public Prosecution Service became an important actor in enforcing the right to health, as seen in the debate reported in the press: “The prosecutor argues that it is the duty of the Public Prosecution Service to make this type of decision because health is a right for everyone and a duty of the state. ... The decision was taken because the body was contacted several times by Paravidda ... with complaints about the shortage of hospital beds for patients with HIV” (Portador…, 3 maio 2003, p.6).

Luiz Antônio da Silva, a leading figure in Paravidda, summed up his memory of these struggles, as well as some coping strategies adopted in a delicate social and health situation. According to him, “the only hospital that served us was Barros Barreto ... So we would go to the secretary to ask for more beds because of the spike in AIDS cases. When it didn’t work ... we would go to the press ... to force the secretary to meet our demands ... We used the press and the press used us” (Silva, 22 set. 2021, p.9-10).

The enforcement of the standards issued by the Ministry of Health often went hand in hand with the occupation of institutional spaces for social participation in SUS. One case in point was Paravidda, which was offered a seat on the Belém Municipal Health Council as of 2001 (Silva, 2011). The council was key for voicing the health demands of people living with HIV in Belém, as reported by Maria Laurinda: “We had a chair. ... It was a battle! Because in these council meetings, the secretary was often present. Then when he was there, the demands were greater” (Correia, 16 set. 2021, p.12).

SUS, created “top-down” through federal laws and regulations, was taking shape at the grassroots level in municipalities and regions in the midst of struggles and according to the local technical, financial, and institutional resources available. In Pará, as in other parts of Brazil, the assurance of access to healthcare for people living with HIV was one of the arenas in which this battle was played out, whose successes, contradictions, and limitations have been largely addressed in this text.

## Final considerations

The years immediately following the resumption of democratic rule in Brazil were challenging. Overall, the federal governments were aligned with neoliberalism, which stood against the creation of a health system that would meet everybody’s needs as a citizen’s right. However, they encountered huge social pressure, which was channeled through a whole gamut of social movements that gradually developed into fully fledged political actors in the defense of their specific interests.

This article investigated some key actors from Pará who, in line with national forces formed in the midst of the challenges of the AIDS epidemic, gradually organized themselves technically and politically with a view to attaining their goals. Throughout the 1990s, they voiced a range of demands in different forums, which influenced how the country’s new public health system was ultimately implemented.

In this context, Paravidda became an influential collective force in its advocacy for the health needs of people living with HIV in Belém. Notwithstanding the differences among its members, one point that rallied unanimous support was the pressing need for more hospital care to treat the complications associated with HIV/AIDS. During the 1990s, as we have seen, healthcare for people living with HIV gradually took shape in response to Paravidda’s strategic actions in municipal seminars, at the regional RNP+ meeting, and in repeated use of the press to publicize their complaints.

So it was that in the first ten years of implementation of SUS, Paravidda came to wield great influence in institutional discussions and movements for policies that would guarantee the right to health for people living with HIV in Belém – something that remains a challenge to this day. The experience of HIV/AIDS activism in Pará therefore indicates that the creation of SUS should not be understood merely as an administrative process, as defined in the Organic Health Laws and subsequent secondary legislation. In a sense, it was quite the contrary: the political struggles, organized in different regions and municipalities, set the political agenda and foundations for the right to health beyond its enshrinement in law. By understanding the creation of SUS as part of the constitution of the Brazilian democratic state, we have revealed that the civil society organizations that championed AIDS-related demands were also instrumental in the construction of this decentralized, democratic, universal health system.

## Data Availability

Not deposited in a data repository.

## NOTES

1 Acquired immunodeficiency syndrome (AIDS) is a disease caused by the action of a virus called human immunodeficiency virus (HIV) (Perlongher, 1986).

2
*Diário do Pará*, part of a communications group headed by Jader Barbalho, was first published in 1982. In the 2000s, the newspaper took a political stance against *O Liberal*, competing for sales with the Maiorana Group and growing political expression (Castro, 2012).

3
*O Liberal* was the leading newspaper in Belém in the 1970s, although its sales had declined by the 2000s as *Diário do Pará* had gained popularity. The Maiorana family, who owned *O Liberal*, was therefore a major political player in the region, making its support felt during election campaigns in Pará (Castro, 2012).

4 Maintained at the Pará Department of Health library, in Belém. More information at: http://www.saude.pa.gov.br/etsus-pa/.

5 The Arthur Vianna library’s periodicals archive is maintained in Belém. Contact details: +55 91 3202 4332 and cbpav@fcp.pa.gov.br.

6 The literature indicates the existence of a significant asymmetry between the parties to the pact established under the terms of NOB-93 (Arretche, 2005; Scatena, Tanaka, 2001).

7 Municipal health authorities, classified as incipient, could provide services but had no autonomy to manage the healthcare network; those classified as partial also had authorization to manage existing public outpatient facilities and to formulate and execute disease surveillance actions; and those classified as semi-full had responsibility for administering the whole local health network except for the state health facilities (Brasil, 20 maio 1993).

8 When a municipality was granted full management of the municipal health system, this meant they were in charge of planning, managing, and evaluating the whole municipal health system: primary care facilities, specialized facilities, and hospitals (Brasil, 5 nov. 1996).

9 When a municipality was granted full management of primary care, this meant they were in charge of planning, managing, and evaluating all the municipality’s primary care facilities (Brasil, 5 nov. 1996).

10 The event was held in Belém on March 13-15, 1998, under the banner “Taking Our Space.” According to the sources interviewed, it was attended by around 70 people (Encontro..., 16 mar. 1998; Portadores..., 4 mar. 1998).

11 Considered a barrier to development, AIDS was incorporated into the World Bank’s goals in the 1990s (Mattos, Júnior, Parker, 2001). The agreements, called AIDS I and AIDS II, provided funding for NGO projects, for disease prevention, for services, and for institutional development, surveillance, research, and evaluation (Barros, 2018; Cueto, Lopes, 2023).

12 This agreement lasted from 1999 to 2002 and involved 165 million dollars from the bank and 135 million dollars from the Brazilian government to expand care for people living with HIV and help improve municipal and state management capabilities (Barros, 2018).
