# Supplementary material for: HIV/AIDS activism and the Unified Health System in Belém, Brazil, 1990-2003
Source: Hist Cienc Saude Manguinhos. 2025 May 19;32:e2025022. doi: 10.1590/S0104-59702025000100022en (PMC12091861; doi:10.1590/S0104-59702025000100022en)
Supplement: Translation

# Translation: O ativismo HIV/aids e o Sistema Único de Saúde em Belém do Pará, 1990-2003

**DOI:** 10.1590/S0104-59702025000100022

**Authors:** Paulo Henrique Souza dos Santos, Carlos Henrique Assunção Paiva

**Affiliations:** i Doutorando, Programa de Pós-graduação em História das Ciências e da Saúde/Casa de Oswaldo Cruz/Fiocruz. Rio de Janeiro – RJ – Brasil paulohen.rique@hotmail.com; ii Coordenador, Departamento de Pesquisa em História das Ciências e da Saúde/Casa de Oswaldo Cruz/Fiocruz. Rio de Janeiro – RJ – Brasil carlos.paiva@fiocruz.br

**Keywords:** História da saúde, Sistema Único de Saúde (SUS, Ativismo HIV/aids

## Abstract

O artigo analisa, em perspectiva histórica, o papel das organizações sociais no enfrentamento da aids como parte do processo de construção institucional do Sistema Único de Saúde (SUS) no estado do Pará, Brasil. Conferindo destaque às atividades do grupo Paravidda, discutem-se as reivindicações pela organização da assistência hospitalar a partir da regulamentação do SUS em 1990 até 2003, quando há uma reestruturação da assistência à saúde na região. Conclui-se que o Paravidda se converteu em um ator de destaque na implementação do SUS na região, significando, assim, que a sociedade civil organizada desempenhou um papel-chave para o processo de reforma da saúde e de construção institucional do SUS.

A partir da publicação das chamadas Leis Orgânicas da Saúde (leis 8.080 e 8.142), em 1990, inicia-se formalmente a implantação do Sistema Único de Saúde (SUS) no país com o objetivo de viabilizar o direito universal à saúde do cidadão brasileiro, de forma descentralizada, integral e com ampla participação popular. Tais princípios e orientações não eram novos, pois estavam nas discussões e lutas de diversos segmentos da sociedade nos anos 1970 e 1980, com destaque para os movimentos sociais, como o movimento sanitário, o movimento popular em saúde e o movimento feminista (Gerschman, 2004; Escorel, 2012).

O processo de implementação do SUS, no entanto, articulando diferentes interesses institucionais e movimentos sociais, revelou-se desafiante nos anos 1990 (Faleiros et al., 2006; Gerschman, 2004; Paim, 2008). A vigência de um ideário neoliberal e, mais que isso, agendas programáticas de governos alinhadas ao neoliberalismo sinalizavam a falta de apoio às políticas sociais que se pretendia implantar. Com inegáveis impactos na saúde, esse cenário político-ideológico configurou um importante obstáculo para o avanço do processo de reforma sanitária (Paiva, Teixeira, 2014).

Colocava-se, de forma evidente, que o SUS não era institucionalmente assegurado, e sua regulamentação, em 1990, anunciava um novo ciclo de embates sociais, aliás, como o próprio processo de luta política em torno da regulamentação já havia demonstrado (Faleiros et al., 2006; Gerschman, 2004; Paim, 2008). Nesse caso, a existência de uma combativa e articulada plenária da saúde foi um componente essencial no enfrentamento aos vetos do presidente Fernando Collor de Mello à lei n.8.080/90, que garantia a implantação dos colegiados de gestão para a participação da sociedade, como os conselhos e as conferências nas três esferas de poder.

Nesse contexto de formulação e implementação do SUS, na primeira década da retomada democrática brasileira, a aids^1^ impulsionava fortes debates sobre sexualidade e saúde a partir do impacto da doença na comunidade homossexual brasileira (Aguiar Júnior, 2016; Daniel, Parker, 2018; Dias, 2012; Nascimento, 2005; Ramos, 2016; Santos, 2019; Vitiello, 2009). Na década de 1990, homens homossexuais, mulheres lésbicas, mulheres prostitutas, bissexuais, travestis, profissionais de saúde, organizaram grupos para formar consensos em relação aos estigmas da doença e à falta de atenção à saúde para as pessoas com HIV positivo, encaminhando, em diversas partes do país, debates e iniciativas alinhadas ao processo de implantação de um sistema de saúde universal.

Na cidade de Belém, Pará, a aids também gerava discussões sobre os estigmas e o acesso à saúde na opinião pública (Santos, 2019). Os jornais *Diário do Pará* ^2^ e *O Liberal* ^3^ tornaram-se espaços estratégicos das organizações não governamentais (ONGs)/aids paraenses na divulgação de eventos e denúncias sobre a situação de saúde. Em plena alvorada do SUS, o Movimento Homossexual de Belém (MHB), Grupo de Mulheres Prostitutas do Estado do Pará (Gempac), Grupo de Apoio e Prevenção do doente de Aids (Gapa-Pa) e o Grupo Para a Valorização, Integração e Dignificação do Doente de Aids (Paravidda) passaram a compor uma rede de luta por atenção à saúde das pessoas com HIV positivo como um direito no estado.

Nesse quadro, os integrantes do Paravidda, entre outros aspectos da saúde, pautavam a necessidade de ampliação de leitos para tratamento dos agravantes clínicos da aids e a organização da rede assistencial pública. Em um cenário de implementação do SUS, essas reivindicações nos fazem perceber o grupo como um dos atores coletivos na construção de assistência à saúde universal em Belém.

A despeito da diversidade de correntes, enfoques e abordagens da historiografia da aids no país, chama a atenção o fato de o SUS ser praticamente tangenciado nas narrativas (Aguiar Júnior, 2016; Barata, 2006; Barros, 2018; Galvão, 1997; Ramos, 2016; Santos, 2019; Souza, 2014; Teodorescu, Teixeira, 2015; Vianna, 2018). Quando mencionado, o sistema de saúde se apresenta como um processo dado e estabelecido e, na prática, um fenômeno paralelo à própria aids no país. Neste texto, pretende-se estabelecer conexões entre o processo de implantação do SUS e as diferentes formas como a sociedade civil organizada, com foco nos movimentos em apoio às pessoas com HIV positivo, posicionou-se em favor do direito à saúde.

Considerando esses elementos, o artigo tem o objetivo de analisar as reivindicações por assistência hospitalar do Paravidda, na cidade de Belém/PA, no contexto da primeira década de implantação do SUS até o ano de 2003, quando ocorre a publicação da portaria n.03/2003, que refere a liberação de novos leitos para a aids no Pará. Atuante a partir da década de 1980, e formalizado em 1992, o grupo foi um dos pioneiros na pauta do acesso à saúde das pessoas que viviam com HIV positivo em Belém.

Para subsidiar a nossa análise, partimos de um pressuposto teórico que considera a esfera de formulação de políticas públicas não restrita aos atores governamentais, mas também sujeita às interferências do amplo conjunto da sociedade. Nesse sentido, a sociedade civil é considerada um lugar de onde surgem necessidades, por vezes, alinhadas a grupos organizados com estratégias para comunicar demandas ao interior de um Estado que se propõe democrático (Bobbio, 2007).

A partir dessa perspectiva, convocamos três conjuntos de documentações. O primeiro se refere aos Relatórios de Gestão da Secretaria Estadual de Saúde do Pará (Sespa),^4^ coleção que identifica as principais iniciativas governamentais na saúde local no alinhamento às Normas Operacionais Básicas do SUS (NOB), que passam a definir os critérios para uma atenção à saúde descentralizada e regionalizada.

Já um segundo conjunto de documentos, envolvendo artigos do jornal *Diário do Pará* e *O Liberal*,^5^ permitiu a identificação de diferentes perspectivas nos debates sobre aids, considerando aspectos inerentes aos periódicos, como os interesses dos veículos de comunicação e dos atores políticos a eles vinculados (Luca, 2005). Nessa coleção, foram consideradas as reportagens em que o Paravidda comunicava denúncias às autoridades locais de saúde e as repercussões dos debates sobre aids em âmbito local. Aqui, a imprensa serviu como estratégia de encaminhar demandas políticas do grupo Paravidda ao interior das instituições oficiais de saúde.

Por fim, um terceiro conjunto, constituído por depoimentos de atores-chave, introduz tensões e perspectivas ausentes nos demais documentos, pois abrange múltiplas subjetividades e olhares dos depoentes sobre as trajetórias aqui contempladas (Voldman, 2006). Os depoentes participaram de entrevistas semiestruturadas e, em geral, tinham dois perfis: ativistas das questões em HIV/aids vinculadas ao Paravidda no período que é objeto desse estudo; e sujeitos vinculados à Sespa e à Secretaria Municipal de Saúde e Meio Ambiente de Belém (Sesma). É importante sinalizar que a pesquisa está aprovada no Comitê de Ética em Pesquisa da EPSJV/Fiocruz, sob o número 4.970.385.

O artigo se organiza em três partes. Na primeira, convocamos o cenário de movimentação das autoridades locais de saúde no processo de construção do SUS no estado. Em seguida, discutimos a formação do ativismo HIV/aids no Brasil e no Pará, bem como a construção político-institucional do Paravidda. A partir desse ponto, exploramos as reivindicações do grupo por assistência hospitalar às pessoas com aids como parte do processo de construção da saúde universal, descentralizada e participativa, pilares organizacionais e doutrinários do SUS.

## Vias institucionais: a construção do Sistema Único de Saúde em Belém do Pará nos anos 1990

Os desafios para a implantação do SUS encontram-se razoavelmente registrados na literatura brasileira (Arretche, Marques, 2002; Cordeiro, 2004; Klein, 2011; Paim, 2008, 2009; Paiva, Fonseca, 2015; Silveira, Paim, Adrião, 2022). Envolvendo diferentes questões e problemas, esse momento inicial tratou da institucionalização da operação do sistema de saúde de forma descentralizada, em direção aos municípios, e unificada, uma vez que se estabelecia comando único em cada esfera de governo. A unificação da assistência individual curativa, então de responsabilidade do Ministério da Previdência e Assistência Social, e a saúde pública, com ênfase na prevenção, sob o comando do Ministério da Saúde, revelou-se um desafio institucional de grande monta e central para o êxito do processo de reforma sanitária. A partir dos anos 1990, sob o amparo das chamadas Leis Orgânicas da Saúde, o desafio brasileiro era implantar esse sistema unificado nas unidades federativas, inclusive no estado e nos municípios paraenses que apresentavam suas próprias dificuldades.

O Pará é o segundo maior estado ao Norte do Brasil, com área de 1.248.042km^2^ e representando 14,66% do território brasileiro (Pará, 2023). Ele compõe a região amazônica, área de cenários socioambientais heterogêneos e multifacetados, compostos por densos centros urbanos, cidades isoladas, diversidade de populações tradicionais e grandes distâncias entre certos municípios, algumas delas a mais de novecentos quilômetros das capitais, que implicam dificuldades para o acesso de boa parte da população aos serviços de saúde (Garnelo, 2019; Kadri, Schweickardt, 2016; Oliveira, 2008; Nunes, 2018). Além disso, as administrações costumam desconsiderar a hidrografia regional e priorizar o transporte rodoviário como única forma de vencer os grandes distanciamentos geográficos (Garnelo, 2019).

As demandas da construção de uma rede de assistência à saúde para a aids se processavam entre as questões do território e a dinâmica de reorganização administrativa da Sespa e da Sesma (Pará, 1991a, 1991b, 1992, 1994, 1997, 1999, 2000). Com os inúmeros interesses políticos e pressões sociais, a reestruturação das instituições locais de saúde se encaminhava no compasso das NOB, que impunham, a partir da capacidade indutora do Ministério da Saúde, o ritmo da reforma da saúde possível (Arretche, 2005; Levcovitz, Lima, Machado, 2001; Scatena, Tanaka, 2001). A construção do Centro de Atenção à Saúde em Doenças Infecciosas Adquiridas (Casadia), em Belém, na segunda metade dos anos 1990, situa-se nesse contexto.

Alinhadas ao governo do então presidente Fernando Collor de Mello, as NOB-91 e 92 foram as primeiras normas de encaminhamento da descentralização em direção aos municípios, um processo político supostamente afinado à reforma sanitária. No entanto, elas estabeleciam regras para os repasses de recursos conforme a capacidade instalada e a produção de serviços hospitalares e ambulatoriais, beneficiando as cidades com melhor infraestrutura de saúde. Não à toa se tornaram objeto de críticas por parte de especialistas e movimentos sociais ligados à saúde, pois não só ampliavam a desigualdade no processo de instalação do SUS, como também destinavam baixos recursos às secretarias estaduais para as correções destas desigualdades (Costa, Silva, Ribeiro, 1999; Levcovitz, Lima, Machado, 2001; Scatena, Tanaka, 2001; Santos, Merhy, 2006).

Inserida nessas mesmas contradições, a Sespa iniciou os esforços para coordenar os municípios na perspectiva da descentralização do SUS. Na gestão estadual de Jader Barbalho (1991-1994), a secretaria também passou a indicar ações para construção de espaços de participação social e assessoria às secretarias municipais, constituindo a Divisão de Municipalização do Estado, em março de 1994, com vistas a enquadrar as estruturas locais de gestão ao que determinava a NOB-93 – SUS (Pará, 1992, 1994).

Essa NOB, por sua vez, estabelecida durante a gestão Itamar Franco (1992-1995), procurou responder às demandas por maior autonomia político-administrativa dos gestores locais do SUS.^6^ Dessa forma, a norma conferia diferentes enquadramentos assistenciais para os entes federados: condição incipiente, parcial e semiplena para os municípios; parcial e semiplena para os estados, todas relacionadas ao grau de complexidade de atividades e funções que os gestores locais assumiriam^7^ (Costa, Silva, Ribeiro, 1999; Levcovitz, Lima, Machado, 2001; Scatena, Tanaka, 2001).

Esses enquadramentos exigiam condições técnicas e administrativas compatíveis às secretarias municipais e à secretaria estadual. Segundo avaliação realizada na gestão estadual de Almir Gabriel, do Partido da Social-Democracia Brasileira (PSDB), havia altos índices de morbidade, insuficiência de materiais básicos, aparelhos e equipamentos médico-hospitalares; deterioração das instalações físicas e carência de profissionais, principalmente em cidades do interior do Pará. Assim, o governo do estado chamava a atenção para a necessidade de maiores articulações entre as instituições estaduais e municipais de saúde com o intuito de organizar o sistema local de saúde em consonância com as diretrizes do SUS (Pará, 1997).

A partir da segunda metade da década de 1990, como parte da agenda de reforma, a pasta estadual responsabilizava-se pela assistência especializada de referência e pelos programas considerados estratégicos. Esse plano também convocava reformas nos centros regionais da Sespa, que ficariam encarregados pelo suporte ao processo de descentralização de municípios sob sua administração. Ademais, registra-se um planejamento em direção à transferência dos serviços básicos para a gestão dos municípios (Pará, 1997).

A Sesma de Belém não seguia caminho distinto. A partir da vigência das NOB-93 e 96, passou a registrar um visível processo de fortalecimento gerencial. A cidade passa, então, a ser enquadrada para a gestão semiplena, em 1996, e Gestão Plena do Sistema Municipal de Saúde (GPSM),^8^ em 1998, tendo, a partir daí, segundo os documentos oficiais, importante papel na gestão e oferta de serviços dos sistemas municipal e estadual de saúde (Pará, 1997, 1998). Entre debates e tensões no governo do presidente Fernando Henrique Cardoso, a NOB-96 entrou em vigor em 1998 e direcionou possibilidades para os municípios assumirem apenas a Atenção Primária se fossem habilitados na Gestão Plena da Atenção Básica (GPAB),^9^ ou todo o sistema de saúde municipal se fossem habilitados na GPSM (Brasil, 5 nov. 1996).

O vigor do processo institucional de implantação do SUS em Belém e no estado do Pará se revela, contudo, menos exitoso quando observamos a imprensa periódica local, que dá conta de inúmeros problemas e conflitos. O jornal *Diário do Pará*, especialmente a partir de 1997, durante a gestão do prefeito de Belém Edmilson Rodrigues, do Partido dos Trabalhadores (PT), acumulou queixas com relação ao que seria um mau funcionamento das Unidades Básicas de Saúde da cidade e do Hospital Pronto Socorro de Saúde Municipal (Secretário..., 2 fev. 1997; Pronto-socorro..., 4 mar. 1997; UMS..., 11 jan. 1997).

A imprensa local registrava embates importantes entre a Sesma e a Sespa envolvendo a construção do Hospital Municipal do Bairro do Guamá, que, segundo os planos do prefeito, desafogaria parte da demanda por alta complexidade da região (Secretário..., 21 out. 1998). Se, de um lado, acumulavam-se denúncias sobre o sucateamento da assistência hospitalar municipal, de outro, chamava-se a atenção para a necessidade de um pacto efetivo que encaminhasse a descentralização tal como prevista pelo SUS (Municipalização..., 5 ago. 1997; Secretário..., 1 jan. 1997; Sesma..., 7 jan. 1997).

Considerando-se os dois conjuntos documentais, a imprensa e os textos oficiais, podemos localizar, em linhas gerais, dois períodos na trajetória da reorganização administrativa das instituições locais paraenses na implantação do SUS. Tudo indica que a Sespa avançou na estruturação, em termos técnico e administrativo, para assessorar os municípios no processo de descentralização entre 1992-1996. A partir de 1997, a Sesma de Belém iniciou sua reorganização para assumir unidades básicas, hospitais municipais e planejar a ampliação da cobertura assistencial da cidade, inclusive para a aids.

Diante das mais variadas carências financeiras e de infraestrutura, bem como as inúmeras disputas e tensões relativas ao processo de partilha de poder, sobretudo quando envolvem políticos e estruturas partidárias concorrentes, podemos considerar que a região avançou no contexto de implantação do sistema de saúde, em boa medida pelo caráter indutor e normativo do Ministério da Saúde, pelas ações administrativas da Sespa e da Sesma. Tal processo, contudo, como veremos a seguir, contou com o protagonismo da sociedade civil organizada, demandando serviços assistenciais e se mobilizando politicamente em um contexto jurídico e político-institucional em que a saúde se apresentava, pela primeira vez, como direito de cidadania no Brasil.

## O ativismo HIV/aids: a constituição do Paravidda em Belém nos anos 1990

A epidemia de aids lançou desafios para os gestores da saúde em seus diferentes níveis (Barros, 2018; Kadri, Schweickardt, 2016; Marques, 2002). De um lado, estava-se diante de uma doença cujo conhecimento clínico era ainda incipiente e as ações terapêuticas, em boa medida, ineficazes. De outro, ela ensejou leituras sociais que associavam a sua causa como algo intrínseco de pessoas categorizadas como “promíscuas”, assim construindo estigmas a grupos marginalizados, tal como homossexuais, travestis, bissexuais, mulheres em prostituição (Dias, 2012; Gonçalves, 1989; Nascimento, 2005; Saldanha, 2023). Essa característica dificultava ações de saúde no campo da aids, uma vez que a doença era publicamente encarada como problema individual em detrimento de ações de saúde pública como qualquer outra enfermidade.

Em meio a esse contexto, diversas organizações sociais foram se estabelecendo para reivindicar atenção à saúde para pessoas com HIV positivo e desmistificar os estigmas. Essas entidades, que seriam denominadas de ONGs-aids, tinham múltiplas finalidades: “... o ativismo político, que reivindicava os direitos dos pacientes, e o da solidariedade, que buscava redefinir os princípios pelos quais a sociedade abordava as pessoas infectadas pelo vírus” (Grangeiro, Laurindo da Silva, Teixeira, 2009, p.90).

A primeira casa de apoio para pessoas acometidas com aids foi fundada pela ativista travesti Brenda Lee em São Paulo, que passou a acolher travestis e outros indivíduos em vulnerabilidade social em função da discriminação referente à aids, à sexualidade e ao gênero, ainda na década de 1980 (Teodorescu, Teixeira, 2015). Ademais, diversos grupos atuaram em defesa dos direitos das pessoas com HIV positivo entre os anos 1980 e 1990, tal como os Grupos de Apoio de Prevenção à Aids (Gapa), a Associação Brasileira Interdisciplinar de Aids, com destaque a Herbert de Souza, e o Pela Vidda, criado por Herbert Daniel, em 1989 (Barros, 2018; Contrera, 2000; Dias, 2012; Green, 2018; Vitiello, 2009; Zaquieu, 2006).

As entidades passaram a organizar reuniões para discutir e alinhar posições sobre atenção à saúde, como os Encontros Nacionais de ONGs-Aids (Galvão, 1997; Ongs/aids, 1989, 1990). Entre múltiplas pautas, os encontros também levantaram divergências, sobretudo em relação à representação de pessoas com HIV positivo por aquelas sem o vírus. Essa questão, dada sua complexidade, levou à formação da Rede Nacional de Pessoas Soropositivas (RNP+), em 1995, que no início questionou o trabalho e a legitimidade das ONGs-aids em função de burocratização e insuficiências (Câmara, Lima, 2000).

Ainda que em meio a tantas tensões, essas articulações, em diálogo com médicos, cientistas e figuras instaladas na burocracia do Estado, foram relevantes para a construção do Programa Nacional de Aids. Tão importante quanto a formalização de uma política, as atuações e articulações das ONGs-aids colaboraram decisivamente para a construção de agendas programáticas, a implementação efetiva de ações, políticas e para a própria produção de respostas concretas aos problemas vividos pelas pessoas com HIV positivo, com destaque para a conquista da distribuição universal de medicamentos por meio do SUS, a partir de 1996 (Barros, 2018; Teodorescu, Teixeira, 2015).

Durante a implantação das Políticas Nacionais e a construção de diversos grupos ativistas pelo Brasil, a Divisão de DST/Aids do Pará foi constituída também contando com a presença da sociedade organizada, com destaque para a atuação do Paravidda, que esteve em diversos espaços de discussões para exigir assistência hospitalar universal em Belém, princípio basilar do sistema nacional de saúde em processo de instauração.

Além do Paravidda, outros grupos participavam das discussões envolvendo as problemáticas suscitadas pela aids na capital paraense, tal como o Gempac e o Gapa-PA. O primeiro foi construído sob liderança de Lourdes Barreto, nos anos 1990, ativista importante na fundação da Rede Brasileira de Prostitutas na década de 1980 e na luta dos direitos das mulheres, principalmente daqueles relacionados às mulheres prostitutas do Pará e do Brasil, dos anos 1990 aos dias atuais. O Gempac é um espaço coletivo que formou consensos sobre as necessidades de mulheres prostitutas paraenses e também sobre educação sexual e prevenção nas arenas de construção de políticas para HIV/aids no estado, tal como o conselho estadual e municipal de saúde (Barreto, 2023; Saraiva, 2009; Teodorescu; Texeira, 2015). Em relação ao Gapa-PA, o grupo foi idealizado nos anos 1980, na Universidade Federal do Pará, composto por homossexuais, estudantes e profissionais de diversas áreas para promover a conscientização da população paraense em relação à aids, diminuir os estigmas e lutar pela garantia de serviços de saúde na cidade (Martins, dez. 1990).

Nesse processo, o Paravidda se formou em reuniões na Unidade de Referência em Aids (URE-Aids) para compartilhar informações sobre o HIV e apoiar pacientes de Belém no correr dos anos 1980. O nome foi conferido pelos integrantes na década seguinte, sendo Augusto Marques, Luiz Antônio da Silva e Maria Laurinda Silva Correia pessoas importantes para a constituição e manutenção do grupo (Correia, 16 set. 2021; Silva, 22 set. 2021; Paravidda, 1992a, 1992b).

Perdas, dores e esperanças uniram esses personagens em torno da causa. O falecimento de um filho em decorrência da aids, por exemplo, foi propulsor para a trajetória de Maria Laurinda na construção do Paravidda. Em suas palavras: “Eu cheguei até o Paravidda porque eu tive um filho de criação e ele foi passar um tempo fora e ... ele veio contaminado pra casa .... Então eu comecei a participar, estudar muito, saber o que era ...” (Correia, 16 set. 2021, p.1).

Para o homossexual Luiz Antônio, o resultado positivo para HIV o levou a uma pequena reunião de apoio na URE-Aids, apontado por ele como o início do grupo. A cada 15 dias, nas dependências do Hospital Universitário João de Barros Barreto, ele compartilhava experiências da vivência com o vírus diante de um conjunto de pessoas (Silva, 22 set. 2021).

É preciso reconhecer, assim, que os integrantes passaram por experiências distintas com o HIV até a formalização do Paravidda. Se Maria Laurinda foi sensibilizada a partir da experiência dolorosa com seu filho, Luiz Antônio carregava o vírus e os estigmas sociais a ele relacionados. A multiplicidade dessas vivências resultou, segundo os seus criadores, em algumas tensões internas ao próprio grupo (Correia, 16 set. 2021; Silva, 22 set. 2021).

De acordo com as assinaturas da Ata da primeira assembleia oficial, realizada em 27 de janeiro de 1992, dez nomes constavam na reunião: Luiz Antônio da Silva, Maria Laurinda Silva Correia, Célia Maria Mattos da Cunha, Valdeliz Matos Teófilo, Ângela Maria Torres da Costa, Jorge Evandro de Amorim Matos, Silvia Helena Silva do Nascimento, Oswaldo Mattos, Jocélio Jorge e José Carlos Nascimento (Paravidda, 1992b).

Em 1994, eles conseguiram se estabilizar em um espaço do governo estadual no bairro do Jurunas, zona periférica de Belém, com a proposta de acolher apenas crianças com HIV positivo abandonadas (Creche…, 15 nov. 1994). Além disso, também passaram a desenvolver trabalhos assistenciais para um público mais amplo e abrigar temporariamente pessoas do interior do estado em recuperação clínica ou para realização de exames médicos (Paravidda, 1992c; Teodorescu, Teixeira, 2015). No final da década, o grupo passou a se destacar paulatinamente nas discussões e demandas por assistência hospitalar e na construção de um programa estadual para o enfrentamento da aids.

## O Paravidda e a assistência hospitalar: a luta por leitos em Belém e a construção institucional do SUS, 1992-2003

Como vimos, a extensão do território paraense e as características demográficas e geográficas específicas lançam inúmeros desafios para o processo de implantação de políticas públicas. No que se refere estritamente à aids, essas condições impunham dificuldades de acesso à assistência hospitalar, notadamente concentrada no Hospital Barros Barreto, em Belém. Diante disso, a construção das políticas de assistência hospitalar para a doença se apresentava como uma questão especialmente penosa.

Espaços como os seminários municipais de aids, a partir de 1992, tornaram-se fundamentais. Ao reunirem representantes de Sespa, Sesma, Divisão Estadual de Aids, Comissão Municipal de Aids, diretores de hospitais e também representantes das ONGs nos debates sobre a doença, os seminários se converteram em lugares de concertação técnico-política e, em especial, de vocalização dos interesses e demandas sociais organizadas. Nesse campo, o Paravidda enxergou a possibilidade de discutir as demandas de saúde das pessoas com HIV positivo na perspectiva do direito à saúde via dois eixos: de um lado, pressionando pela extensão dos serviços de saúde, e, de outro, pela implementação de ações de prevenção à doença.

Nos dois primeiros seminários, realizados em 1992 e 1993, o Paravidda se ateve à questão da assistência à saúde, considerada muitíssimo restrita. O grupo, junto com outros atores, empenhou-se especialmente na defesa da construção de um hospital onde as pessoas pudessem passar o dia, na garantia de leitos em outros hospitais conveniados ao SUS e no atendimento em nível ambulatorial, contando com a aquisição de medicamentos em quantidade suficiente (Comissão…, 5 dez. 1992).

Ainda que a imprensa divulgasse avanços na assistência à aids em Belém, na segunda metade dos anos 1990, a insatisfação expressiva de gestores e ativistas sobre a situação da capital continuava. A partir de 1997, por exemplo, com a eleição do prefeito Edmilson Rodrigues, do PT, Helena Brígido é designada para a Coordenação Municipal de Aids em Belém. Segundo ela, existiam problemas na operação do Serviço de Atendimento Especializado (SAE): “No ano que eu fui pro SAE ..., ele era minúsculo em relação à URE-Aids. E o ambulatório de aids funcionava lá. ... A gente não tinha espaço e era mais difícil internar pacientes” (Brígido, 3 out. 2021, p.3). Incômodos a respeito do funcionamento desse serviço também passaram pelo depoimento de Luiz Antônio do Paravidda: “... a parte de baixo era uma Unidade Básica de Saúde e a parte de cima para a aids, mas a recepção era única ... nós passamos a discutir isso. Nós precisávamos de um espaço melhor” (Silva, 22 set. 2021, p.8).

Considerando tais desafios, a estratégia do Paravidda para estimular o debate público sobre ampliação da assistência hospitalar foi direcionada a outro espaço na segunda metade dos anos 1990. O primeiro Encontro da RNP+ do setor Norte^10^ foi organizado por uma comissão composta por Antenor Chaves, Jaime Siqueira, Joaquim Barbosa e Luiz Antônio da Silva, com o objetivo de promover intercâmbio de informações entre as pessoas com HIV positivo do Norte do Brasil (Portadores..., 4 mar. 1998; Portadores..., 20 mar. 1998). Esse evento reuniu integrantes de Amazonas, Amapá, Acre, Rondônia, Roraima e Pará, assim como a coordenadora municipal de aids, Helena Brígido, trabalhadores da saúde, representantes de Paravidda, Gempac, Gapa-PA e MHB. O jornal *O Liberal* destacou um resumo do evento, indicando questões pontuadas por Luiz Antônio sobre a ampliação da assistência à saúde e as dificuldades categorizadas como próprias da região amazônica. Esses aspectos foram sinalizados no trecho a seguir:

Luiz Antônio da Silva diz que o portador do vírus HIV que mora na Amazônia não possui os mesmos privilégios do portador residente nas regiões Sul e Sudeste. A começar pelo tratamento dispensado pela Sespa ... Como nos últimos dois anos registrou-se um crescimento ... no número de casos ... torna-se necessária ... mais uma unidade de referência especializada em Aids (Encontro…, 16 mar. 1998, p.3).

É nesse contexto que se dá, sob a liderança de Helena Brígido, o processo de implementação do Casadia, finalizado em 1999, como uma iniciativa da coordenação municipal, em diálogo com ONGs e o apoio do Programa Nacional de Aids. Nas palavras de Brígido (3 out. 2021, p.3): “Eu escrevi o projeto Casadia ... Eu fui em Brasília, ... para várias reuniões. ... Nós tínhamos reuniões frequentes com as organizações não governamentais. ... Gapa, Paravidda, MHB. ... O Paravidda era a Dona Laura [Maria Laurinda]. A gente reunia e pedia para fazer documento para encaminhar para o secretário”.

A reformulação do SAE e sua ampliação para o ambulatório municipal Casadia, em 1999, também foi favorecida pela implementação dos recursos do Banco Mundial^11^ por meio do Aids II,^12^ via Programa Nacional de Aids, e pelo fortalecimento das instâncias de saúde locais. Nesse sentido, destaca-se que Belém já tinha se adequado à categoria de gestão semiplena do SUS, em 1996, e à GPSM, em 1998, aspectos que sinalizam acúmulos de capacidade institucional para a Secretaria Municipal de Saúde expandir seus serviços. Como gestor semipleno, o município passava a reunir formalmente uma série de competências, entre elas “a titularidade sobre autorização de credenciamento, descredenciamento, controle e avaliação dos serviços ambulatoriais e hospitalares privados e filantrópicos; gerenciamento da rede ambulatorial” (Costa, Silva, Ribeiro, 1999, p.39). Essa característica do município como principal coordenador dos serviços do sistema municipal também foi reforçada no enquadramento à GPSM, em 1998.

A inauguração do Casadia, portanto, no final da década de 1990, é parte de um processo que, não apenas envolveu articulações entre gestores e movimentos sociais, como também representou um inegável fortalecimento institucional de estruturas executivas e prestadoras de serviços de saúde na região. Diante de conquistas e ganhos institucionais, a imprensa local registrava, nesse mesmo período, que o Hospital Barros Barreto continuava sendo a única estrutura de assistência à saúde de atendimento às pessoas com HIV positivo durante as noites e aos finais de semana (Vem…, 22 set. 1999; Casa-dia…, 27 out. 1999).

Situações como essa demonstravam que a resposta às necessidades das pessoas com HIV positivo dependia, decisivamente, do suporte oferecido pelas ONGs, conforme sinalizou Maria Laurinda Correia (16 set. 2021, p.11), quadro do Paravidda: “Se tivesse com paciente ruim, mandava ele pra Casadia e ele passava o dia todo lá. A ambulância vinha buscar. Passava o dia todo lá, no soro e médico. Pela noite a Casadia mandava pro Paravidda e de manhã cedo ele voltava”.

Ademais, também demonstravam que a implantação de um sistema único de saúde, com base no direito, não era algo certo, simples e desprovido de contradições. As incertezas impostas ao processo, pela vigência de governos e de doutrinas liberais, e também de dificuldades políticas e institucionais, era contrabalançada, no que se refere à resposta ao problema da aids, pela articulação de três aspectos favoráveis: uma articulação nacional de ONGs-aids, o fortalecimento do Programa Nacional de Aids e o processo institucional de descentralização e participação social do SUS, que conferiu múltiplas possibilidades de articulações em apoio ao próprio sistema de saúde.

Tal como noticiou o jornal *O Liberal* em de março de 2002, as denúncias e pressões por melhorias na assistência à saúde, inclusive contando com recursos judiciais, encontravam amparo na Constituição de 1988: “Entre elas, a exigência de que os estabelecimentos disponibilizem um determinado número de leitos para atendimento desse público. ... já que o artigo n.196 da Constituição Federal estabelece como dever do Estado e direito de todo cidadão a garantia de acesso universal e igualitário às ações e serviços de saúde” (Falta…, 5 mar. 2002, p.7).

Um problema reiterado em muitas outras situações e matérias jornalísticas, e que ao longo do tempo estimulou conexões entre o Paravidda, o Ministério Público, a OAB e o Gapa-PA, diz respeito à luta por melhorias no tratamento hospitalar das pessoas com quadros clínicos de aids, cujo êxito estava constantemente incerto e flutuante. Em novembro de 2002, por exemplo, o mesmo jornal publicou um informe sobre novos leitos em Belém a partir de 2003: “Os pacientes portadores de aids vão poder ser atendidos em mais dois hospitais de Belém, a Santa Casa e o hospital das clínicas, que estarão disponibilizando leitos a partir de março de 2003” (Santa…, 8 nov. 2002, p.5). Sem dúvida, uma conquista

foi alcançada após uma longa luta de três anos da ONG Paravidda junto ao Ministério Público e Ordem dos Advogados do Brasil. A diretora do Paravidda, Maria Laurinda Corrêa, disse que há três anos ela procurou o Ministério Público para denunciar que os hospitais não atendiam. ... ‘Pedimos também que descentralizassem do Barros Barreto o atendimento, porque essa era uma necessidade dos doentes’ (Santa…, 8 nov. 2002, p.5).

No contexto do início dos anos 2000, as demandas dos movimentos sociais, retratadas nas matérias dos jornais, passaram a se inscrever nas discussões em torno da regionalização da saúde e na organização da rede assistencial, tal como sinalizado pelas Normas de Assistência à Saúde (Noas), de 2001 e 2002. Essas normas ensejavam esforços na promoção de maior integração regionalizada da saúde nos estados federados, considerando as desigualdades de acesso à assistência (Trevisan, Junqueira, 2007; Pereira, Quito, 2004).

As Noas, no seu conjunto, atribuíram funções aos executivos estaduais no desenvolvimento de ações de planejamento regional da rede, tanto de modo a racionalizar o uso dos recursos existentes quanto de melhor prover o acesso aos serviços por parte da população. Para tal, o Plano Diretor de Regionalização (PDR) seria o recurso de gestão para organizar os estados brasileiros em módulos assistenciais, definindo polos e referências assistenciais. Em última instância, os municípios deveriam trabalhar de forma integrada em relação à assistência à saúde, e cada polo de grupo de municípios (módulos assistenciais) se converteria em referência assistencial para um conjunto mínimo de serviços (Trevisan, Junqueira, 2007; Pereira, Quito, 2004).

Diante de um estado como o Pará, caracterizado por mesorregiões marcadas por importantes desigualdades internas e grandes distâncias entre cidades do interior e Belém, o plano buscava diminuir a dependência de municípios menores à capital do estado (Brasil, 1991; Pará, 2003a). Em 2003, ano de elaboração do PDR paraense, não à toa, houve grande expectativa pública em torno da ampliação dos leitos para aids no estado (Saúde..., 3 jul. 2003).

A despeito dos avanços institucionais, na prática, o debate e as demandas com vistas ao acesso à assistência hospitalar continuaram presentes vigorosamente na imprensa local nesse mesmo período (Tratamento…, 14 fev. 2003). Como parte das movimentações sociais, a atuação incisiva das ONGs, com destaque para o grupo Paravidda, esteve frequentemente associada às ações do Ministério Público, com o intuito de ampliar a abertura de leitos na capital e em outras regiões do estado. Tais iniciativas, sob os novos marcos institucionais, implicaram o planejamento de leitos direcionados à aids para os nove polos de saúde que estavam sendo elaborados no PDR (Pará, 2003b).

Os leitos voltados para as complicações da aids seriam implementados se as secretarias municipais encaminhassem o cadastro até o final de março de 2003. Nos municípios em GPSM, como Belém, Ananindeua e Castanhal, os secretários de saúde tornaram-se responsáveis pela identificação dos hospitais. Já nas cidades habilitadas na GPAB, a disponibilidade dos leitos estava sob responsabilidade das unidades hospitalares gerenciadas pelo governo estadual (Pará, 2003b).

A duras penas, a rede de assistência hospitalar do SUS para pacientes vivendo com HIV estava sendo construída no estado da seguinte forma nos nove polos do PDR: no Polo Metropolitano, em Ananindeua com seis leitos e em Belém com 41, sendo quatro no Hospital Barros Barreto. Além disso, dois leitos em Marituba, um em Castanhal e Barcarena; para o Polo Atlântico, um leito no Hospital Regional de Salinópolis; no Polo Guamá, um leito em Paragominas; no Polo Tocantins, um leito em Tucuruí; no Polo Carajás, dois leitos em Marabá; no Polo Xingu, um leito no Hospital regional de Altamira; no Polo Araguaia, um leito em Redenção; para o Polo Tapajós, considerando a característica regional e o acesso, foi pensado em subdividi-lo em duas áreas de referência: dois leitos em Santarém e Itaituba; no Polo Marajó, considerando o acesso para o município de Belém, as internações, bem como um leito, deviam se dar no Hospital Barros Barreto (Pará, 2003b).

Nessa mesma resolução foi indicada a necessidade de planejamento de 62 leitos que, segundo a perspectiva do secretário de saúde Fernando Dourado, não representavam apenas a expansão de leitos em Belém, mas uma “tão esperada descentralização no atendimento desses pacientes” (Tratamento…, 14 fev. 2003, p.8). Ou seja, a necessária construção institucional de uma rede assistencial do SUS na região.

As emergências clínicas, contudo, eram de tal ordem que os avanços institucionais não se revelavam ainda capazes de atender às necessidades dos usuários do SUS. Atentemos, por exemplo, para as frequentes denúncias feitas pelo Paravidda à Sesma sobre a crônica falta de leitos. Maria Laurinda Correia chegou a apontar que pessoas do interior do estado, mas também da capital, buscavam constantemente abrigo e apoio do grupo enquanto aguardavam tratamento. Segundo ela: “Tem pessoas que vieram para cá com chance de se recuperar, mas demoraram a se internar e acabaram morrendo” (Leito…, 29 mar. 2003, p.7). Em resposta, a Sesma relatava repasses de pedidos para o cadastramento de novos leitos à Sespa, a quem cabia o encaminhamento para o Ministério da Saúde oficializar mediante portaria específica (Leito…, 29 mar. 2003).

Diante disso, o Ministério Público se colocava como um importante instrumento para fazer valer o direito à saúde, conforme constatado no debate público na imprensa periódica: “... o promotor argumenta que é dever do Ministério Público fazer esse tipo de determinação por tratar-se da saúde um direito de todos e dever do Estado. ... A decisão foi tomada porque o órgão foi procurado várias vezes pelo Paravidda ... com queixas pela falta de leitos nos hospitais para os pacientes portadores de HIV” (Portador…, 3 maio 2003, p.6).

Luiz Antônio da Silva, uma das lideranças do Paravidda, sintetizou a memória dessas lutas, bem como algumas estratégias de enfrentamento adotadas em um quadro social e sanitário delicado, segundo ele: “... o único hospital que atendia a gente era o Barros Barreto. ... Aí nós íamos ao secretário pedir o aumento do número de leitos por causa do crescimento exagerado dos casos de aids. Quando não dava certo ... a gente ia na imprensa ... forçar o secretário a atender as nossas reivindicações. ... A gente usava a imprensa e a imprensa usava a gente” (Silva, 22 set. 2021, p.9-10).

A utilização e incorporação prática das normas emanadas do Ministério da Saúde se juntavam à ocupação dos espaços institucionais de participação social do SUS. Nesse sentido, merece destaque a indicação de um assento pelo Paravidda no Conselho Municipal de Saúde de Belém a partir de 2001 (Silva, 2011). Essa arena institucional foi fundamental para vocalizar as demandas de saúde das pessoas com HIV positivo em Belém, tal como relatado por Maria Laurinda: “Nós tínhamos uma cadeira. ... Era briga! Porque nessas reuniões de conselho, muitas vezes o secretário tava presente. Aí quando ele tava presente, as cobranças eram maiores” (Correia, 16 set. 2021, p.12).

O SUS engendrado “pelo alto”, por meio de portarias federais, ia se fazendo no chão de terra dos municípios e regiões, a partir das lutas e dos próprios limites técnicos, financeiros e institucionais encontrados em diferentes territórios do país. No Pará, como em outras regiões do Brasil, a garantia do acesso à saúde das pessoas com HIV positivo foi um dos eixos de luta, com êxitos, contradições e limites que foram em boa medida tratados neste texto.

## Considerações finais

Os anos iniciais da retomada da democracia formal no país foram desafiadores. Em regra geral, os governos federais eleitos se alinharam à plataforma neoliberal, frontalmente contrária à implementação de um sistema de saúde que se propunha a atender a todos com base no direito de cidadania. No entanto, as demandas sociais eram imensas, expressavam-se a partir dos mais diversos movimentos sociais e, assim, convertiam-se em forças políticas que pressionavam a estrutura estatal.

Neste texto, contemplamos personagens que, a partir do estado do Pará, e em consonância com forças nacionais formadas em meio às demandas da epidemia de aids, paulatinamente foram se organizando técnica e politicamente com vistas ao alcance dos seus objetivos. Ao longo da década de 1990, eles passaram a vocalizar um conjunto de exigências direcionadas às arenas de discussões da saúde pública, que se articulavam com o processo de implantação de um renovado sistema de saúde no país.

Diante disso, o Paravidda se apresentou como uma força coletiva de lutas e acolhimento às demandas de saúde das pessoas com HIV/aids em Belém. Em que pesem as diferenças internas do grupo, a imediata necessidade de compor um quadro de assistência hospitalar mais ampla para atender às complicações das pessoas que viviam com aids foi o ponto conciliador das posições da entidade. Durante os anos 1990, como vimos, a organização da saúde relacionada à aids tomou forma, como parte das estratégias do Paravidda, nos seminários municipais, no encontro da RNP+ regional e nas recorrentes denúncias nos veículos de imprensa.

É nesse sentido que, na década inicial de implantação do SUS, o Paravidda se configurou como ator social relevante nas discussões e movimentações institucionais para implementar políticas que garantissem o direito à saúde das pessoas com HIV positivo em Belém, o que permanece uma construção desafiadora até os dias atuais. A experiência do ativismo HIV/aids no Pará sinaliza, portanto, que a implantação do SUS não deve ser compreendida como um mero processo administrativo, definido nas Leis Orgânicas da Saúde e nos demais instrumentos normativos subsequentes. Em certo sentido, o processo é inverso. A luta política, organizada em diferentes regiões e municípios, conferiu agenda e base políticas para que o direito à saúde superasse o enunciado formal. Considerando o SUS como parte do processo de constituição do Estado democrático brasileiro, podemos dizer que a sociedade civil organizada em torno das demandas de aids também esteve na construção de um sistema de saúde descentralizado, democrático e universal.

## Data Availability

Não estão em repositório digitais.

## NOTAS

1 A síndrome da imunodeficiência adquirida (sida) ou *acquired immunological deficiency syndrome* (aids) é uma doença causada pela ação de um vírus chamado vírus da imunodeficiência humana **(**HIV) (Perlongher, 1986).

2 O *Diário do Pará* iniciou sua circulação em 1982, interligado ao grupo de comunicação chefiado por Jader Barbalho. Nos anos 2000, o jornal assumiu oposição política contra *O Liberal*, disputando vendas com o grupo Maiorana e crescente expressão política (Castro, 2012).

3 O jornal liderava o mercado em Belém na década de 1970, chegando aos anos 2000 com um decréscimo em função do crescimento do *Diário do Pará*. A família Maiorana, vinculada ao jornal *O Liberal*, destaca-se como “jogadora” política da região, fazendo seu apoio se tornar importante nos processos eleitorais paraenses (Castro, 2012).

4 Localizados na Biblioteca da Sespa, em Belém. Mais informações em: http://www.saude.pa.gov.br/etsus-pa/.

5 O acervo físico da Hemeroteca da Biblioteca Arthur Vianna é situado na cidade de Belém. Contato: (91) 3202-4332; cbpav@fcp.pa.gov.br

6 A literatura sinaliza para a existência de importante assimetria entre as partes no pacto estabelecido por meio da NOB-93 (Arretche, 2005; Scatena, Tanaka, 2001).

7 A condição incipiente enquadrava o município como prestador de serviços, sem autonomia para gerenciar a rede. A gestão parcial atribuía o gerenciamento das unidades ambulatoriais públicas existentes, assumindo a formulação e execução de ações de vigilância. Já na gestão semiplena, o município passava a assumir a administração de toda rede de saúde local, exceto as unidades de referências estaduais (Brasil, 20 maio 1993).

8 A GPSM responsabilizava o município pelo planejamento, gerenciamento e avaliação de todo o sistema municipal de saúde (unidades básicas, unidades especializadas, hospitais) (Brasil, 5 nov. 1996).

9 A GPAB responsabilizava o município pelo planejamento, gerenciamento e avaliação das unidades básicas de saúde (Brasil, 5 nov. 1996).

10 Evento realizado em Belém entre 13 e 15 de março de 1998 com a temática “Assumindo Nosso Espaço”. Segundo as fontes, o encontro reuniu cerca de setenta pessoas (Encontro..., 16 mar. 1998; Portadores..., 4 mar. 1998).

11 Considerada entrave ao desenvolvimento, a aids entrou nos objetivos do Banco Mundial na década de 1990 (Mattos, Júnior, Parker, 2001). Denominados Aids I e Aids II, os acordos destinaram recursos para o financiamento de projetos de ONGs, para prevenção, serviços, desenvolvimento institucional, vigilância, pesquisa e avaliação (Barros, 2018; Cueto, Lopes, 2023).

12 Esse acordo vigorou entre 1999-2002, com 165 milhões de dólares do banco e 135 milhões do governo federal, para ampliar a assistência às pessoas com HIV e para fortalecer as gestões estaduais e municipais (Barros, 2018).
